# Investigation on the Solar Absorption Property of the Nanoporous Alumina Sheet for Solar Application

**DOI:** 10.3390/ma12142329

**Published:** 2019-07-22

**Authors:** Song He, Yanmei Zhang, Wansheng Yang, Zhangyuan Wang, Xudong Zhao, Pingnuo Wang

**Affiliations:** 1 School of Civil and Transportation Engineering, Guangdong University of Technology, Guangzhou 510006, China; 2 School of Materials and Energy, Guangdong University of Technology, Guangzhou 510006, China; 3 School of Engineering, University of Hull, Hull HU6 7RX, UK; 4 School of Architecture and Urban-Rural Plannin, Fuzhou University, Fuzhou 350108, China

**Keywords:** anodic oxidation method, nanoporous alumina sheet, solar radiation, absorptivity

## Abstract

In order to improve the absorption performance of the aluminum sheet for solar application, the nanoporous alumina sheets with the pore diameters of 30 nm and 400 nm were prepared by the anodic oxidation method. The absorption properties of the nanoporous alumina sheets under different solar radiation intensity were studied and compared with the conventional polished aluminum sheet. The results showed that the average absorptivity of the aluminum sheets decreased with the increase of the radiation intensity. When the radiation intensity was 100 W/m^2^, the nanoporous alumina sheet with the 30 nm pore diameter had the highest average solar absorptivity of 0.39, which was 18% higher than that of the nanoporous alumina sheet with 400 nm pore diameter, and 50% higher than that of the polished aluminum sheet. The maximum instantaneous absorption efficiency of the nanoporous alumina sheet with 30 nm pore diameter was found at 0.92 when the radiation intensity was 100 W/m^2^. The testing results indicated that the nanoporous alumina sheet with the 30 nm pore diameter performed the best compared with the other two aluminum sheets. By error propagation analysis, the relative error of the average amount of heat absorption and the average absorptivity were acceptable.

## 1. Introduction

Over the past few decades, the sharp increase in the energy demand has occurred due to global economic development and population growth, leading to rapid consumption of fossil fuels and serious environmental pollution consequences, e.g., air pollution, acid rain, depletion of the ozone layer and global climate change. The development of the renewable energy to replace the traditional energy is imminent. Renewable energy sources (including hydropower, wind, biomass, geothermal, tidal, wave and solar energy sources) can satisfy the energy demands with minor environmental impact compared with the traditional energy sources [1,2,3]. Of which, solar energy, due to it being abundant, cheap and pollution-free, offers a great alternative to conventional fossil fuel resources and is expected to play an increasingly significant role in the global energy future [4,5]. The solar radiation reaching the earth’s surface is approximately 3.4 × 10^24^ J in one year; more than 7500 times the world’s total annual primary energy consumption of 4.5 × 10^20^ J [6]. Therefore, directly and efficiently absorbing solar energy is a significant method to improve the efficiency of solar application systems, e.g., the solar thermal system and PV (photovoltaic) system.

As far as today, vacuum deposition, magnetron sputtering and electro-deposition were the major methods to produce the selective absorber coatings for solar systems [7,8,9,10]. Valleti et al. [11] used the cathodic arc physical vapor deposition technique to prepare the functionally multilayered Cr/CrTiAlN-G/TiAlN/AlSiN/AlSiO coating suitable for enhancing the solar selectivity of the stainless steel substrates for using in the concentrated solar power systems. The results indicated that the optimized coating exhibited the solar absorptivity of 0.95 and thermal emissivity in the range of 0.09 to 0.14 when the ambient environment was up to 600 °C. Gao et al. [12] obtained the spectrally selective solar absorber coating of TiC/Al_2_O_3_ on the stainless steel substrate by the magnetron sputtering method, which performed with the absorptivity of 0.92 and emissivity of 0.13 at 82 °C. Zou et al. [13] prepared the spectrally selective CrAlN-CrAlON coating based the tandem absorber by the magnetron sputtering method. The optimized absorber exhibited the high absorptance of 0.984 and low emittance of 0.07 at 82 °C. Tharamani et al. [14] reported the coatings deposited by the electro-deposition method, and the parameters were optimized to achieve the high solar absorptance of 0.94 and low emittance of 0.08. These methods could achieve the relatively high solar absorptivity, but usually lead to the weakly adhesive materials, which suffered from the degradation over a long period and easily absorbed water and other air impurities, resulting in the degradation of the optical performance of the materials, especially in high temperature and high humidity environments [15,16]. 

In order to resolve the above-mentioned problems of selective absorber coatings, nanoporous materials have been introduced, which can effectively reduce the reflection and scattering of sunlight. Wang et al. [17] obtained the uniquely foamed nanostructure selective absorber coatings comprised a large number of nanoparticle agglomerates and nanopores by facile hydrothermal method on stainless steel. The coatings showed the excellent solar thermal performance with the solar absorptance and thermal emittance of 0.92 and 0.12, respectively. Wang et al. [18] designed the Al-AlN-based selective coatings with the self-assembled silkworm cocoon-like nanostructure. The absorptance of the coating reached the maximum of 0.97, whereas maintaining the low emittance. Cuevas et al. [19] prepared the cobalt pigmented alumina composite coating electrochemically, which performed with the absorptivity of 0.92 and emissivity of 0.16. Galione et al. [20] impregnated Ni into porous alumina onto an aluminum sheet as a substrate by the electrochemical method. This selective surface had an absorptance and emittance of 0.82 and 0.07, which was efficient for solar to thermal energy conversion. Wu et al. [21] prepared porous C-TiO_2_ nanocomposite films by the photopolymerization-induced phase-separation method, and these films show high solar absorptance (α = 0.766–0.863), low thermal emittance (ε = 0.06–0.12). However, nanoporous coatings still had some shortcomings, such as a long manufacturing process and poor controllability of the multi-step process [22].

In this paper, the nanoporous alumina sheets with the pore diameters of 30 nm and 400 nm and pore depth of 100 μm would be prepared by using the anodic oxidation method with the characteristics of stable performance, simple manufacturing, low cost, and high solar absorptivity. Nanoporous alumina sheets with different pore diameter could be obtained by changing the current magnitude. Current density range was 0.4–4.4 A/dm^2^. When prepared nanoporous alumina sheets with surface pore diameter of less than 30 nm, the oxidation current was small, and it was difficult to prepare nanopores with uniform distribution; when prepared nanoporous alumina sheets with surface pore diameter larger than 400 nm, the oxidation current was too large, and it was easy to break down the aluminum sheets. Therefore, the largest diameter nanoporous alumina sheet and the smallest diameter nanoporous alumina sheet were selected. The preparation method would be further improved. In order to investigate the solar absorption property of the proposed nanoporous alumina sheets, they would be compared with the conventional polished aluminum sheet under the different simulated radiations. The instantaneous solar absorptivity, average amount of absorbed heat, and average solar absorptivity would be analyzed, and the correlation of the solar absorptivity with the irradiation would be established. This research would present the advantages of the prepared nanoporous material and indicate the potential application in the solar market.

The purpose of this work is not to develop new materials, nor to optimize the absorption and emissivity. Porous alumina layers with inclusion of transition metals like Fe, Co, Ni, Cu, Au, Ag, Mo, Cr and W have been widely studied [22]. These surfaces have been proven to be highly efficient in converting solar energy into heat. However, there has been little research on a pure porous alumina layer, and most previous porous aluminum layer pore depths were about 1 μm or less [23,24]. This paper aims to study the absorption of solar energy by pure porous alumina layer with a pore depth of 100 μm, highly absorbing surfaces can be obtained by increasing the number of reflections of solar incident ray in the nanopores. Thereby simplifying the preparation process.

## 2. Working Principle of the Nanoporous Alumina Sheet

**Figure 1:**
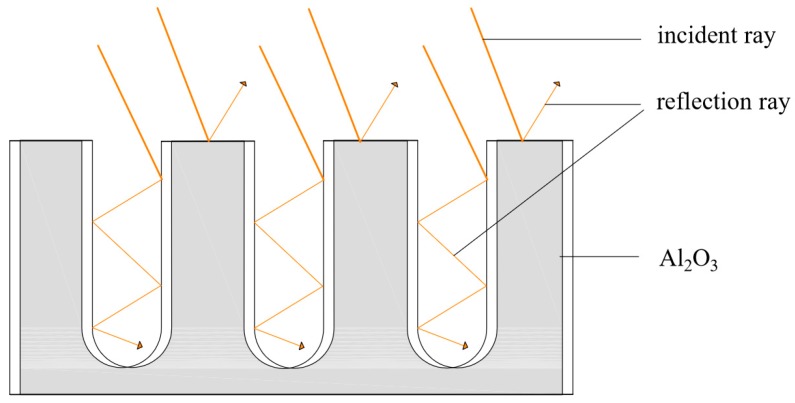
Schematic of the nanoporous alumina sheet.

As shown in Figure 1, the working principle of the nanoporous alumina sheet would be summarized as follows. When the sunlight reaches to the nanopores on the surface of the aluminum sheet, part of them will be absorbed by the aluminum sheet, while the remaining part will be reflected inside the nanopores and eventually absorbed by the aluminum sheet. This process could reduce the reflection of the sunlight from the surface of the aluminum sheet to the environment, and therefore, enhance the solar absorption efficiency. In addition, the multiple-reflecting effects of the nanoporous alumina sheet with small aperture are more obvious. At the same pore depth, the smaller the aperture is, the more times of reflection in the nanopores, and the more sufficient the heat absorption of the aluminum sheet [25].

## 3. Preparation of the Nanoporous Alumina Sheet

The preparation of the nanoporous alumina sheet could be summarized into two steps: (1) Pretreatment of the aluminum sheet to make it smooth, clean and flat; and (2) anodic oxidation process to form the nanopores on the surface of the aluminum sheet. 

### 3.1. Pretreatment

**Figure 2:**
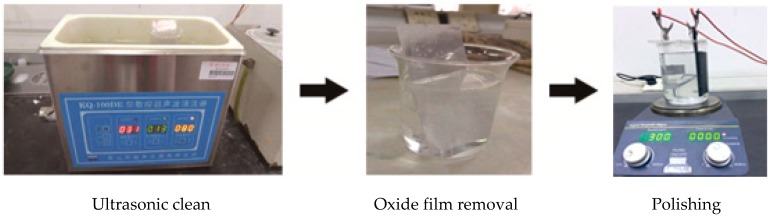
Pretreatment of the aluminum sheet.

The pretreatment process of the aluminum sheet was shown in Figure 2.

The detailed processes could be summarized as follows. Firstly, the aluminum sheets with a purity of 99.99% purchased from Guantai Metal Materials Co., Ltd. (Xingtai, China) was flattened by using the flattening machine (HD-25, Guang’an Haoxin Machinery Equipment Co., Ltd., Langfang, China). Then the aluminum sheet placed in the ethanol/acetone solution was cleaned for 10 min in the ultrasonic cleaner (JP-040, Jiemeng Cleaning Equipment Co., Ltd., Shenzhen, China) to eliminate the oil. After that, the aluminum sheet was placed in the 1 mol/L sodium hydroxide solution (99.6% powder, Shanghai Bio-way Technology Co., Ltd., Shanghai, China) for five minutes to further remove the dirt and completely remove the natural oxide film on the surface to reveal the pure metal matrix, which was to ensure the uniformity of the surface. At last, the aluminum sheet would be polished in the 400 mL perchloric acid/ethanol solution (72%/75%, Shanghai Bio-way Technology Co., Ltd., Shanghai, China) mixed in the ratio of 1:9 by using the aluminum as the anode and graphite (S150100, Beijing Diantan, Beijing, China) as the cathode, and stirring using the magnetic rod (N52, Hongsheng Magnetic Industry, Shanghai, China) for two minutes. 

### 3.2. Anodic Oxidation Process

**Figure 3:**
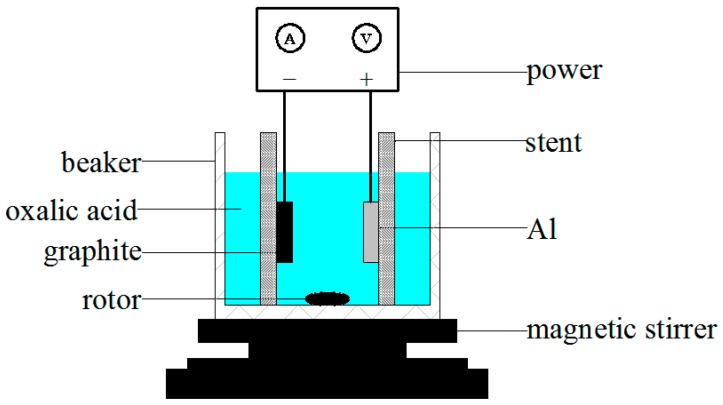
Schematic of the anodic oxidation process of the aluminum sheet.

**Figure 4:**
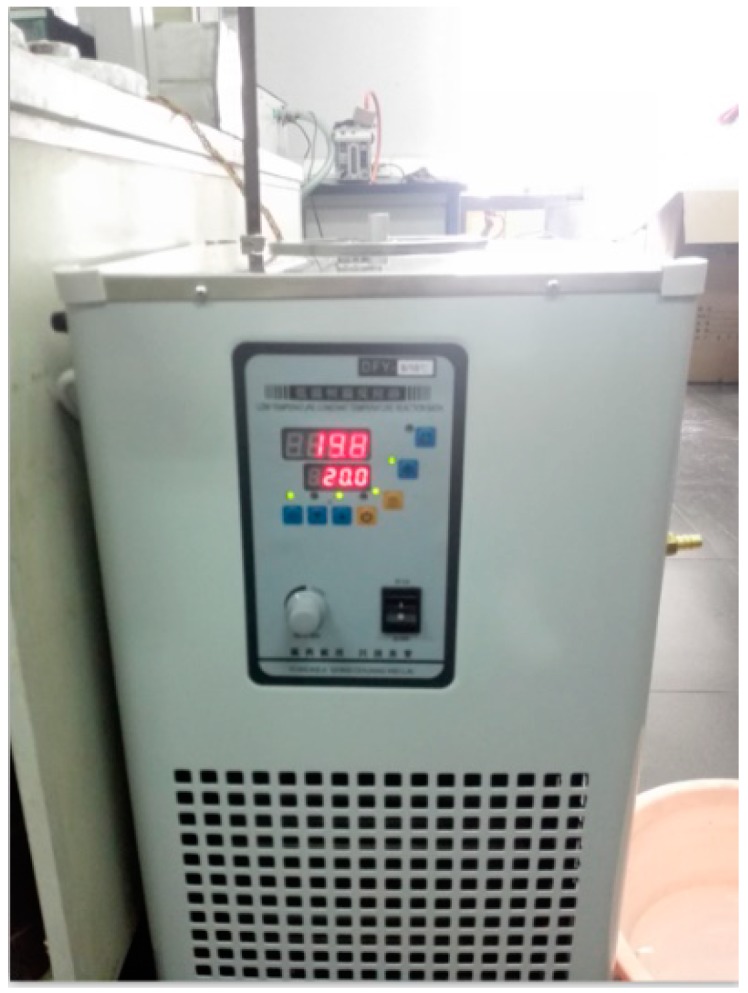
Device of the low-constant temperature.

The schematic of the anodic oxidation process of the aluminum sheet was shown in Figure 3, and this process was carried out in the low-constant temperature device (C4000, Xi’an Xiaxi Electronic Technology Co., Ltd., Xi’an, China) shown in Figure 4, which could provide a constant temperature of 0 °C for the oxidation process of the aluminum sheet, ensuring the uniform distribution of the nanopores on the surface of the aluminum sheet and reducing the current breakdown of the aluminum sheet due to excessive temperature, improving the success rate of preparation of the nanoporous alumina sheet. In this process, the aluminum sheet was taken as the anode, while the graphite was as the cathode. Both would be immersed in the oxalic acid solution (1%, Shanghai Bio-way Technology Co., Ltd., Shanghai, China), and connected to the DC (direct current) power to form the nanopores on the surface of the aluminum sheet, the current was applied in the Ampere. The distance between the aluminum sheet and graphite was at 5 cm, and the process was controlled at the temperature of 0 °C. The magnetic stirrer would be used to make sure the reaction evenly.

### 3.3. Scanning Electron Microscope

**Figure 5:**
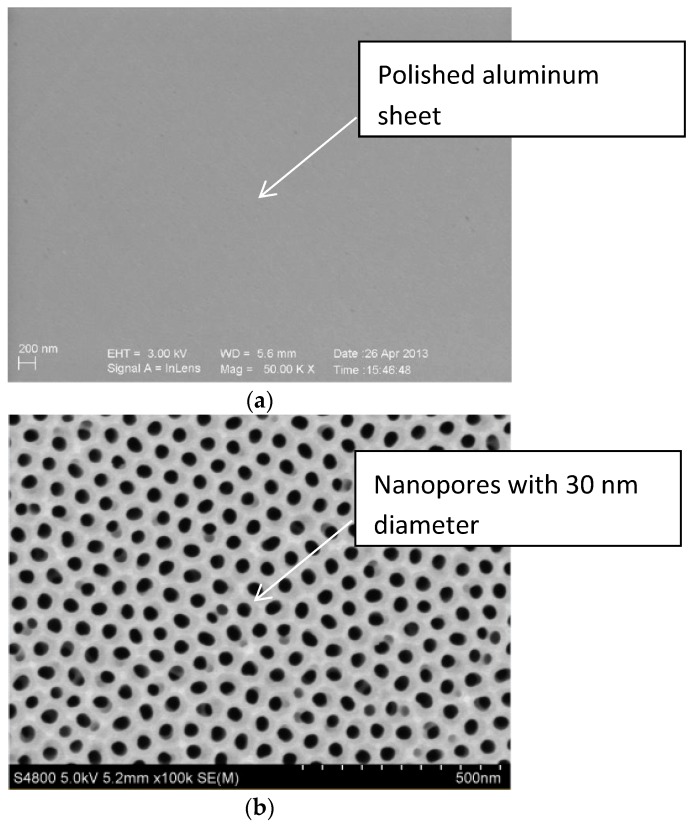

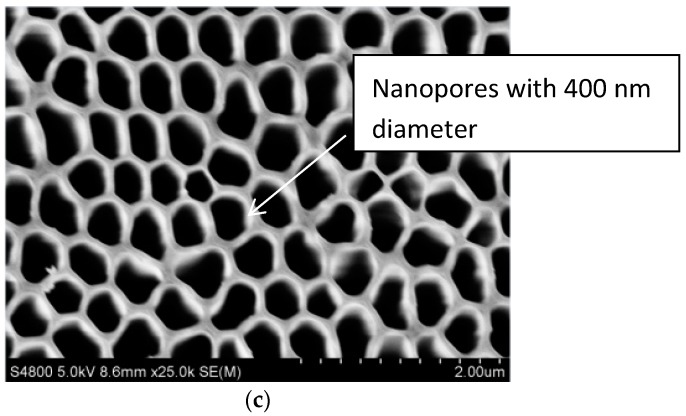
The SEM image of the aluminum sheet. (**a**) polished aluminum sheet; (**b**) nanopores with 30 nm diameter; (**c**) nanopores with 400 nm diameter.

The scanning electron microscope (SEM, S-3400N, HITACHI, Japan) would be used to inspect whether the micro-pores distributed uniformly on the surface of the aluminum sheet, and Figure 5 showed the SEM image of the aluminum sheet. Figure 5a was a polished aluminum sheet for comparison with porous alumina sheets, Figure 5b detected the average pore size of 30 nm when the image magnified 100,000 times, and Figure 5c for the average pore size of 400 nm when the image magnified 25,000 times. Both indicated that the nanoporous alumina sheet performed with the evenly distributed micro-porous structure and consistent aperture.

## 4. Experimental Performance Investigation 

In order to investigate the solar absorption performance of the nanoporous alumina sheet, the aluminum sheets with the pore diameters of 30 nm and 400 nm were prepared using the anodic oxidation method mentioned above and compared with the conventional polished aluminum sheet. It should be mentioned that due to the limits of the budget and experimental conditions, these two typical types of the nanoporous alumina sheet would only be made. Further tests will be conducted for the wide range of the pore diameters of the aluminum sheet.

### 4.1. Construction of the Testing Rig 

**Figure 6:**
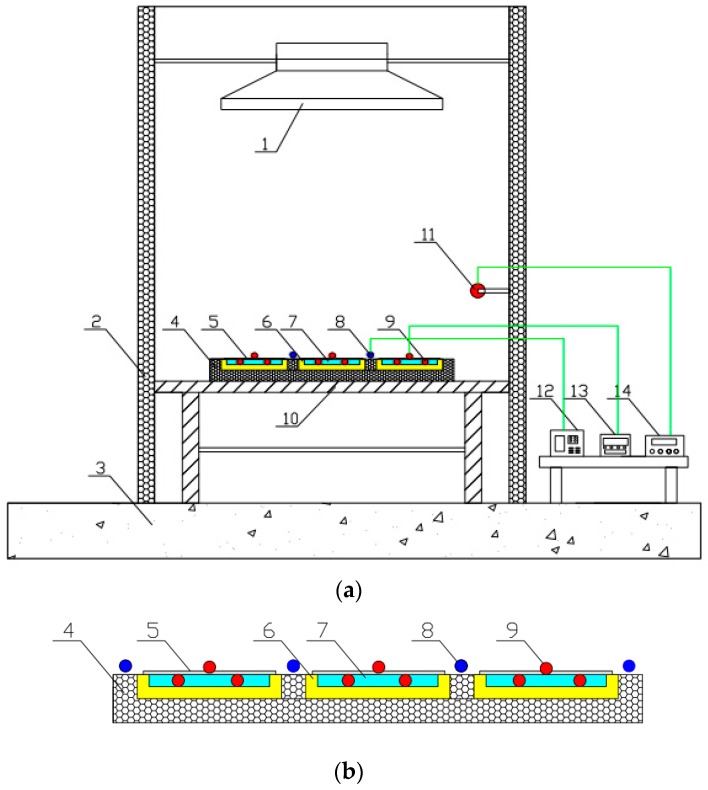
Construction of the testing rig. (**a**) whole construction; (**b**) partial enlargement. 1—solar radiation simulator; 2—baffle plate; 3—floor; 4—heat insulating material; 5—aluminum sheets; 6—water container; 7—water; 8—pyranometer; 9—thermocouples; 10—bracket; 11—temperature/humidity probes; 12—solar radiation observation station; 13—multichannel temperature monitor; 14—multichannel temperature/humidity detection system.

**Figure 7:**
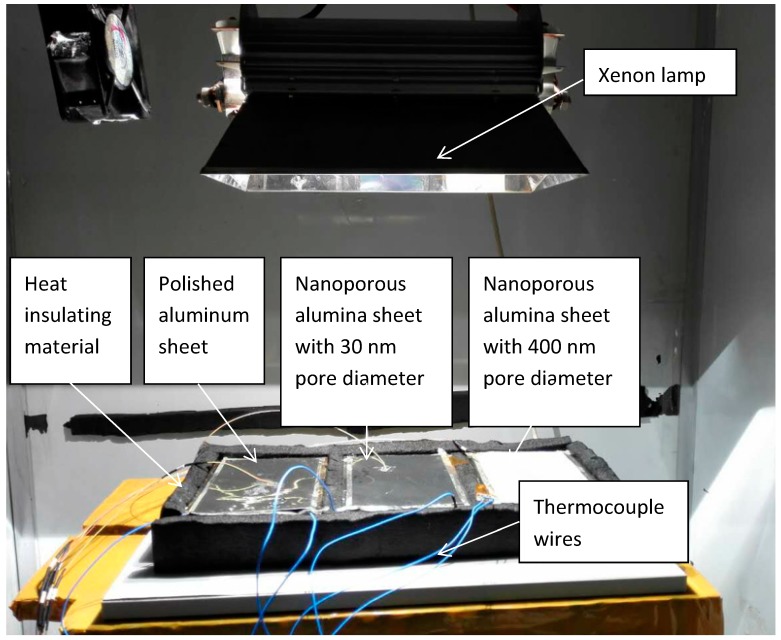
Image of the testing rig.

As shown in Figure 6 and Figure 7, the testing rig was constructed in the laboratory of the Guangdong University of Technology, China. The testing rig included the water container, insulation material, and aluminum sheets (i.e., polished aluminum sheet, nanoporous alumina sheet with the pore diameter of 30 nm, and nanoporous alumina sheet with the pore diameter of 400 nm). The water containers, each sized at 150 mm × 100 mm × 10 mm and filled in with 150 g water, were made of plexiglass. The thermal insulation material, i.e., extruded polystyrene board with the thermal conductivity ranged at 0.04–0.2 W/(m·K), surrounded the water container to prevent the heat losses to the surroundings. The three prepared aluminum sheets, each sized at 150 mm × 100 mm × 0.2 mm and weight at 8 g, were put above the water container to measure the solar absorption and heat transfer from the sunlight to the cold water in the water container through the aluminum sheets.

One xenon lamp was used to simulate the sunlight with the different radiations from 100 W/m^2^ to 800 W/m^2^ through regulating the input voltage of the lamp, and the simulated radiations were recorded by the pyranometer. The solar radiation intensity has been tested around the surface of the container, and their deviations were less than 5%. The testing would be operated in three hours, and the temperatures of the system components, e.g., aluminum sheet and water, were recorded every 20 min. The thermocouple wires transmitted the signal from the temperature measuring points to the temperature monitor. The characteristics of the devices used in the experiments were presented in Table 1.

### 4.2. Analysis and Discussion of the Testing Results

The three parameters determining the solar absorption property of the aluminum sheets, i.e., instantaneous absorptivity, average amount of heat absorption and average absorptivity, would be analyzed and discussed. 

#### 4.2.1. Instantaneous Absorptivity

**Figure 8:**
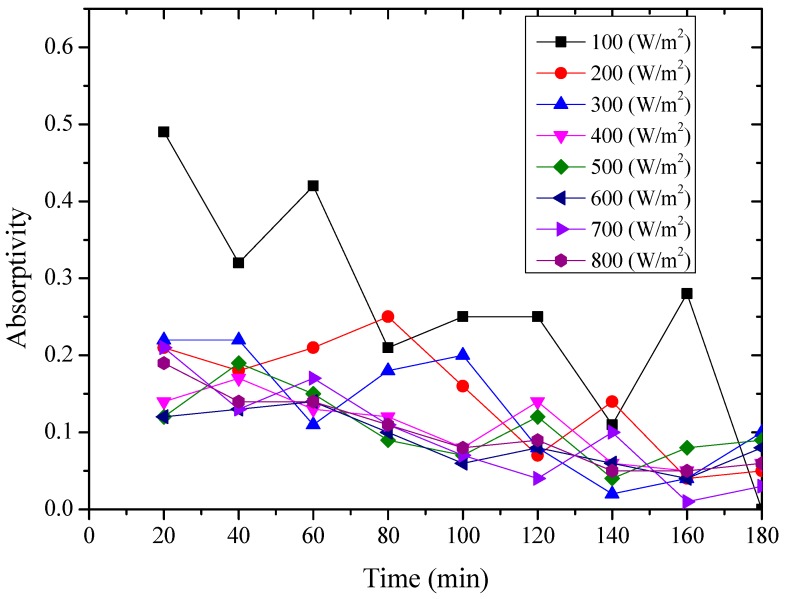
Variation of the instantaneous absorptivity for the polished aluminum sheet.

**Figure 9:**
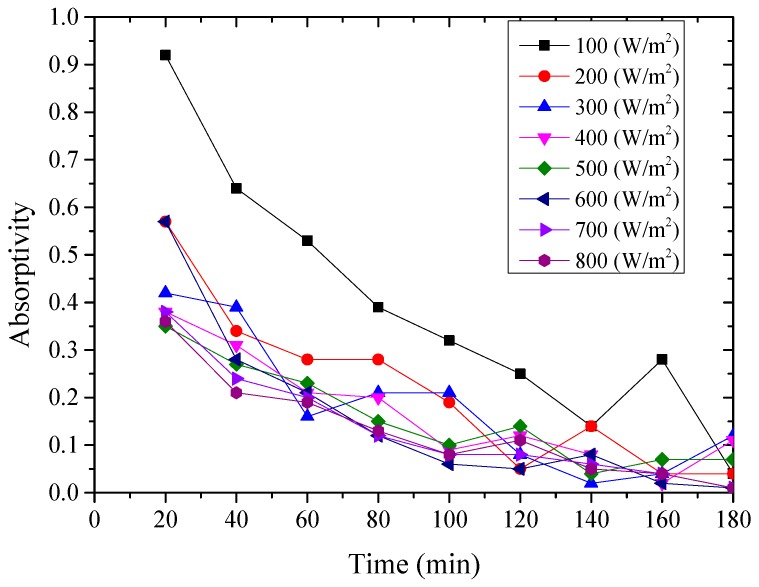
Variation of the instantaneous absorptivity for the nanoporous alumina sheet with 30 nm pore diameter.

**Figure 10:**
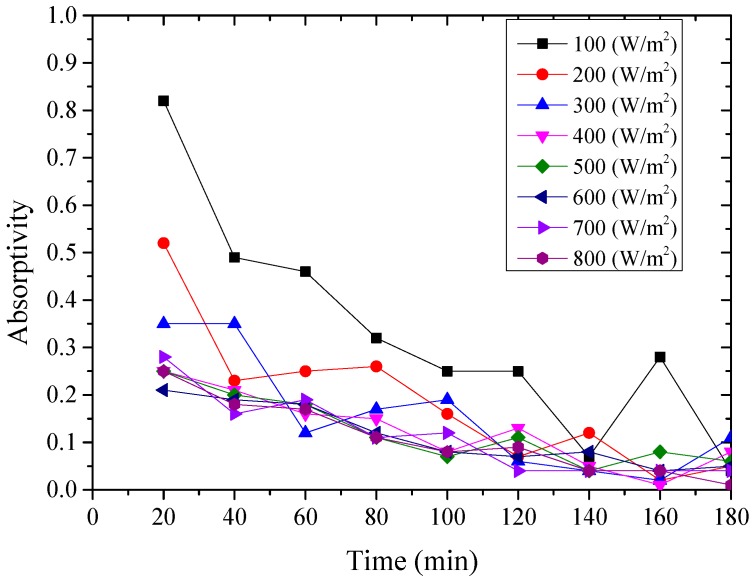
Variation of the instantaneous absorptivity for the nanoporous alumina sheet with 400 nm pore diameter.

The instantaneous absorptivity of the system could be calculated according to Equation (1) [26], and presented from Figure 8, Figure 9 and Figure 10. *Q′*_t_ was the total amount of heat absorbed by the aluminum sheet and water every 20 min, which could be calculated by the change of water temperature and aluminum sheet temperature, due to their mass and specific heat capacity were known.

(1) $$\alpha^{\prime }=\frac{{Q^{\prime }}_{t}}{AI\Delta t^{\prime }}.$$

From above Figure 8, Figure 9 and Figure 10, it could be seen that the variation of the instantaneous absorptivity of the three testing rigs presented similarly that it decreased and then reached stable due to part of the absorbed energy by the aluminum sheet dissipated to the environment. The instantaneous absorptivity of the nanoporous alumina sheet with 30 nm pore diameter decreased significantly than that of the other two testing rigs for the first hour of the testing, since the pore size of this type of the aluminum sheet was much smaller than the other two sheets, leading to much increased heat losses to the ambient and decreased solar absorptivity eventually. The instantaneous absorptivity was also found to be decreased with the increasing of the radiation intensity. When the simulated solar radiation was at 100 W/m^2^, the maximum instantaneous absorptivity of the three testing rigs reached 0.92, 0.82 and 0.49 respectively. 

#### 4.2.2. Average Amount of Heat Absorption

**Figure 11:**
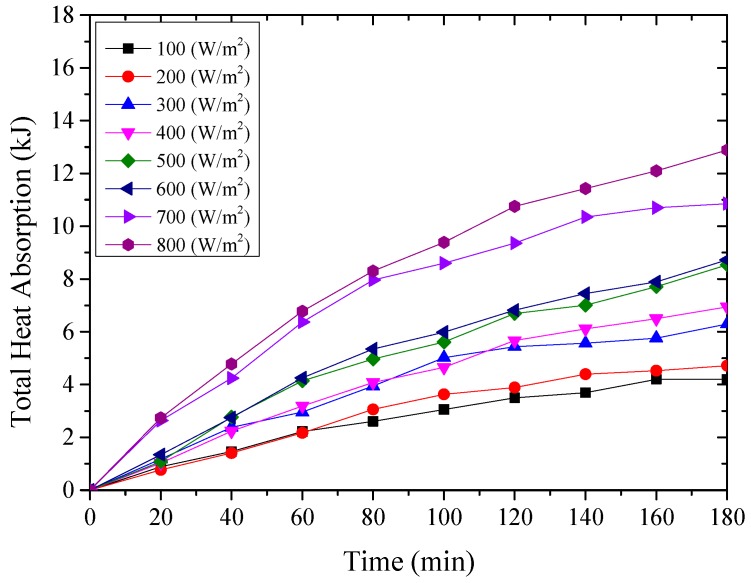
The amount of heat absorbed by the polished aluminum sheet.

**Figure 12:**
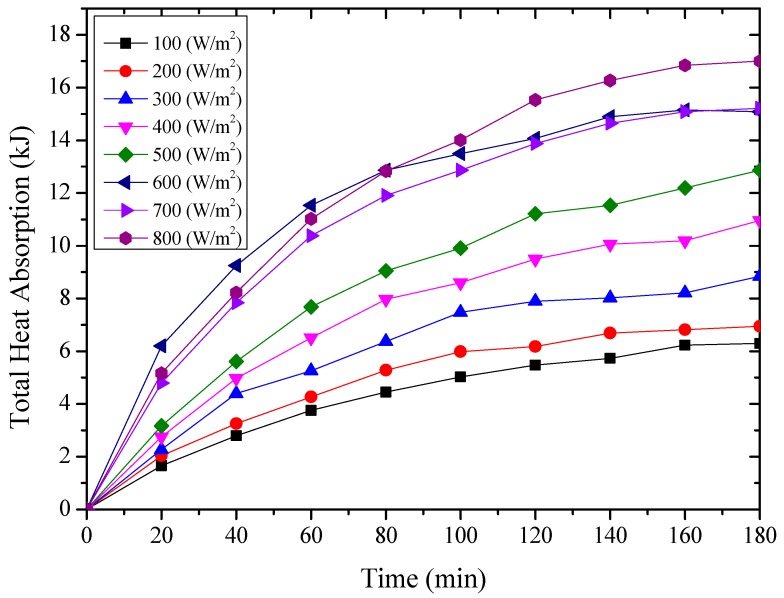
The amount of heat absorbed by the nanoporous alumina sheet with 30 nm pore diameter.

**Figure 13:**
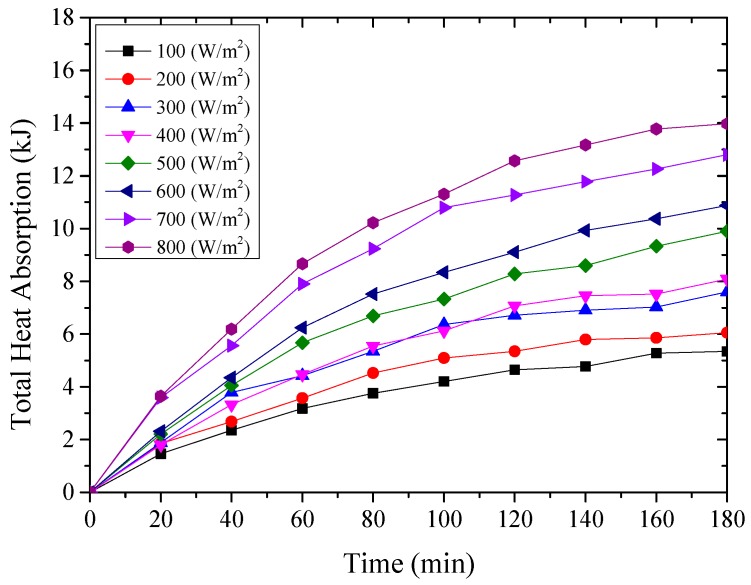
The amount of heat absorbed by the nanoporous alumina sheet with 400 nm pore diameter.

The thermal energy absorbed by the testing rig was composed of the amount of the heat absorbed by the aluminum sheet and cold water inside the water container, which were expressed from Equation (2) to Equation (4) [27], and the results were shown from Figure 11, Figure 12 and Figure 13 for the testing rigs operated under different simulated radiations. It should be mentioned that due to the well thermal insulation of the extruded polystyrene board, the heat losses from the water container to the environment were ignored.

(2) $$Q_{t}=Q_{\mathrm{al}}+Q_{w}$$

(3) $$Q_{\mathrm{al}}=c_{p,\mathrm{al}}m_{\mathrm{al}}\left(T_{\mathrm{al}}-T_{\mathrm{al}0}\right)$$

(4) $$Q_{w}=c_{p,w}m_{w}(T_{w}-T_{w0})$$

From the above Figure 11, Figure 12 and Figure 13, it could be seen that the thermal energy absorbed by the testing rigs increased from the beginning of the test and then tended to be constant. During the start-up stage, the temperature of the aluminum sheet was relatively low, and therefore, the amount of heat absorbed by the aluminum sheet was greater than the radiant heat losses, leading to the increased solar absorption heat of the aluminum sheet. After that, the temperature of the aluminum sheet gradually increased, and the temperature difference between the aluminum sheet and ambient directly increased the radiant heat losses.

It could also be found that for the testing rigs operated under different working conditions, the amount of absorbed heat increased with the increase of the simulated solar radiation. When the simulated solar radiation was at 800 W/m^2^, the heat absorption of the three testing rigs, i.e., the nanoporous alumina sheets with 30 nm and 400 nm pore diameters, and polished aluminum sheet, reached the maximum at 17,003 J, 13,974 J and 12,887 J respectively. This meant that the nanoporous alumina sheet performed better than the conventional polished aluminum sheet on the solar absorption characteristics, and the nanopore size of the aluminum sheet influenced its performance that the aluminum sheet with 30 nm nanopore diameter worked better than the sheet with 400 nm nanopore diameter.

#### 4.2.3. Average Absorptivity

**Figure 14:**
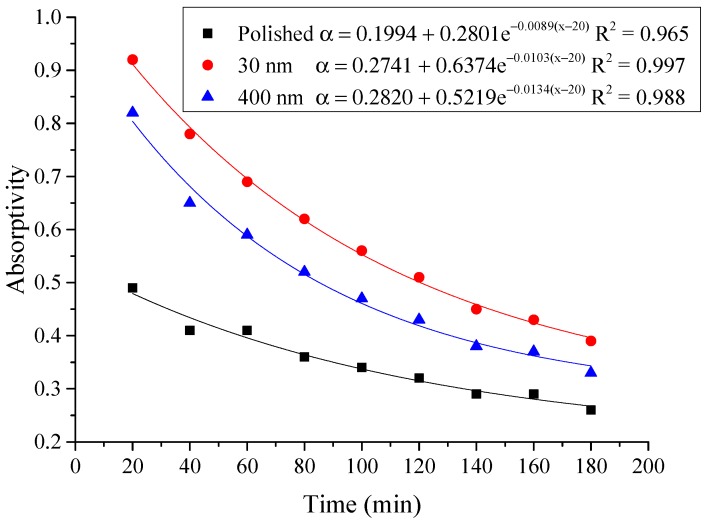
Variation of the average absorptivity for the testing rigs operated under the simulated radiation of 100 W/m^2^.

**Figure 15:**
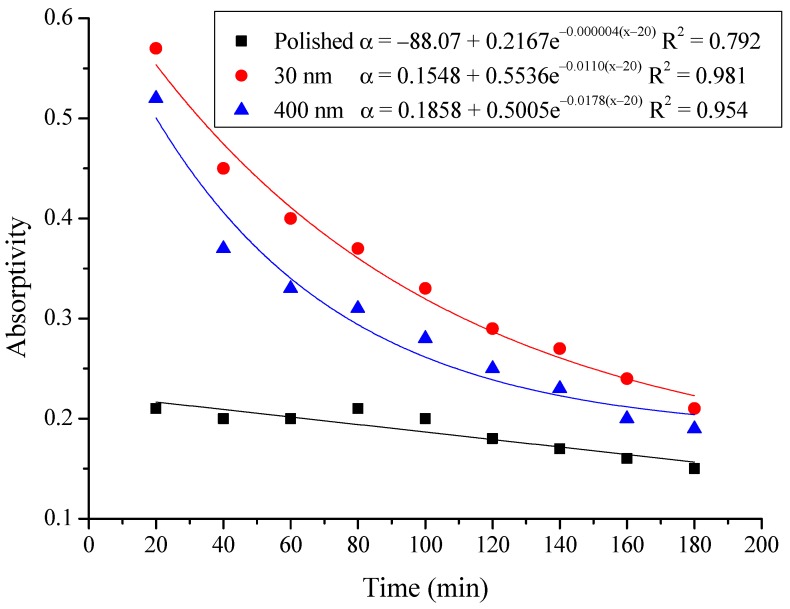
Variation of the average absorptivity for the testing rigs operated under the simulated radiation of 200 W/m^2^.

**Figure 16:**
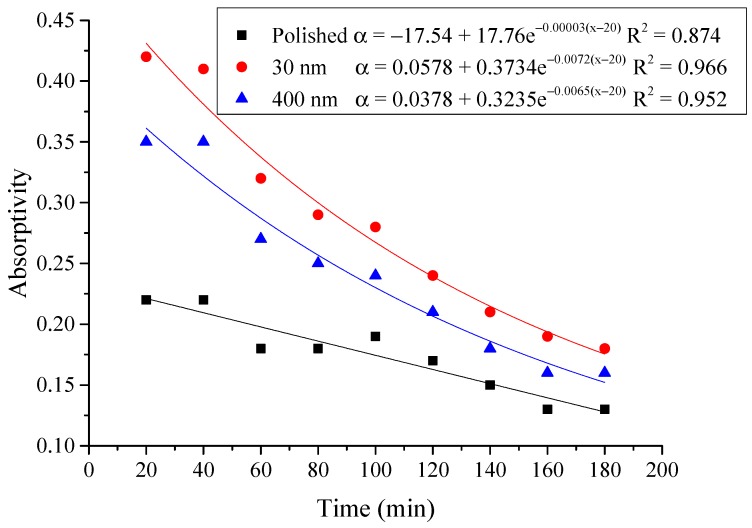
Variation of the average absorptivity for the testing rigs operated under the simulated radiation of 300 W/m^2^.

**Figure 17:**
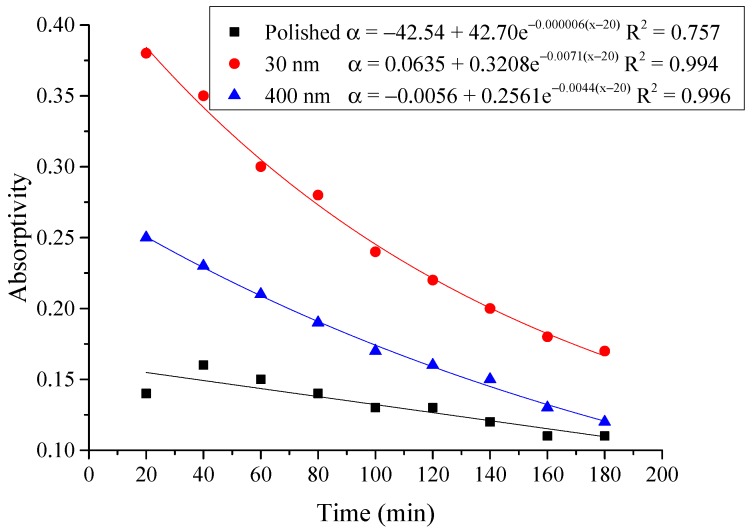
Variation of the average absorptivity for the testing rigs operated under the simulated radiation of 400 W/m^2^.

**Figure 18:**
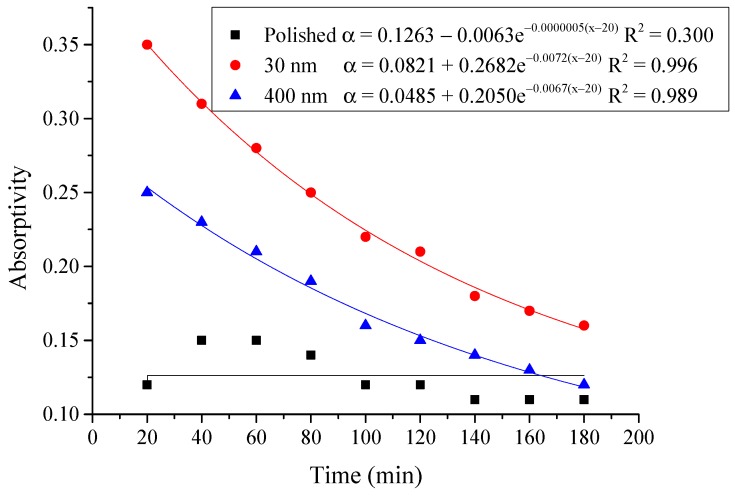
Variation of the average absorptivity for the testing rigs operated under the simulated radiation of 500 W/m^2^.

**Figure 19:**
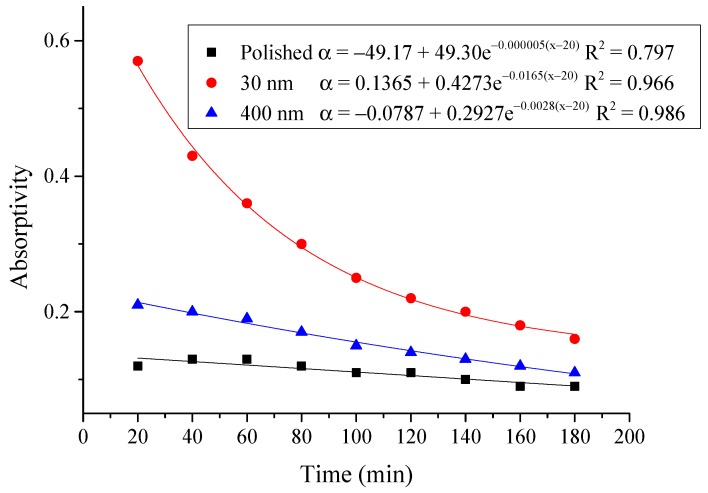
Variation of the average absorptivity for the testing rigs operated under the simulated radiation of 600 W/m^2^.

**Figure 20:**
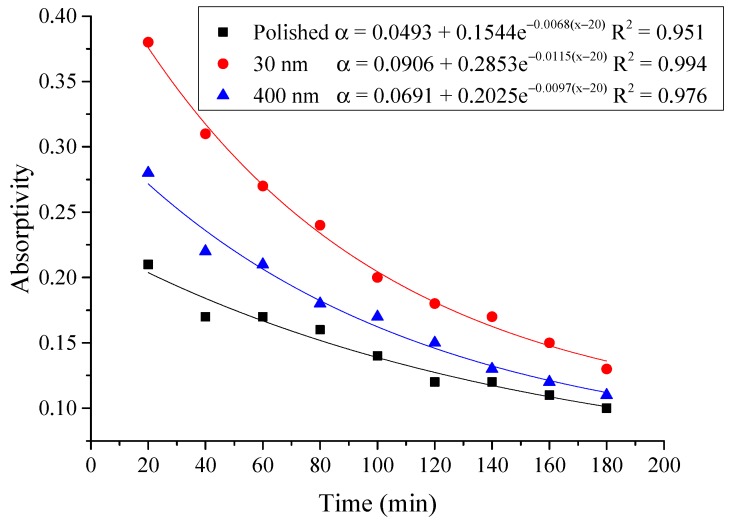
Variation of the average absorptivity for the testing rigs operated under the simulated radiation of 700 W/m^2^.

**Figure 21:**
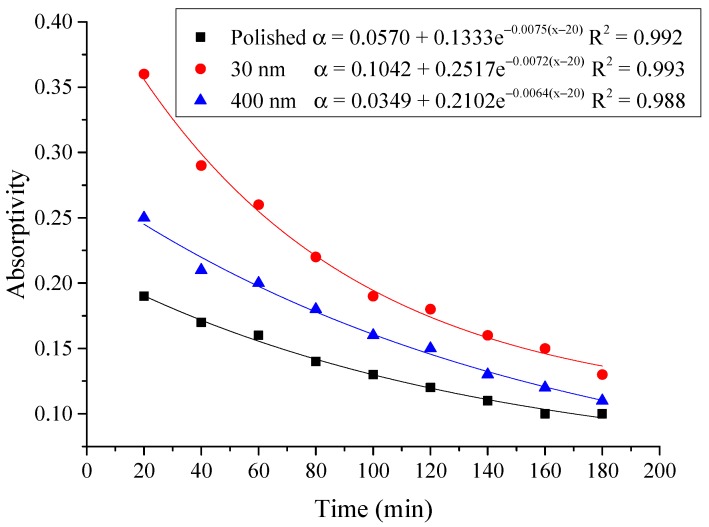
Variation of the average absorptivity for the testing rigs operated under the simulated radiation of 800 W/m^2^.

The average absorptivity was defined as the ratio of the total absorbed radiation by the testing rigs to the total radiation emitted from the solar simulator as in Equation (5), and the testing results were calculated and presented in Table 2 and Figure 14, Figure 15, Figure 16, Figure 17, Figure 18, Figure 19, Figure 20 and Figure 21. *Q*_t_ could be calculated by Equation (2). Δt was the radiation time.

(5) $$\alpha =\frac{Q_{t}}{60\times AI\Delta t}$$

From the above figures and table, the surface absorptivity was found to be decreased with the increase of the radiation intensity, and the average absorptivity of the nanoporous alumina sheet with 30 nm pore diameter reached the maximum at 0.39 when the simulated radiation was at 100 W/m^2^, which was 18% higher than that of the nanoporous alumina sheet with 400 nm pore diameter (0.33) and 50% higher than that of the polished aluminum sheet (0.26). When the simulated radiation was at 800 W/m^2^, the minimum average absorptivity of the three testing rigs was at 0.14, 0.10 and 0.08 respectively. The average absorptivity of the three aluminum sheets was also found to be decreased exponentially with time, and the nanoporous alumina sheets decreased significantly compared with the polished one due to the higher surface temperature of the nanoporous alumina sheets, leading to more radiant heat losses to the surroundings, as shown in the above Figure 14, Figure 15, Figure 16, Figure 17, Figure 18, Figure 19, Figure 20 and Figure 21, the trend of the polished aluminum sheet was relatively flat and tended to be linear.

#### 4.2.4. Correlation of the Absorptivity with the Radiation Intensity

Based on the above test results, the correlation of the absorptivity of the aluminum sheet with the radiation intensity was established in the exponential function.

(6) $$\alpha ={\mathrm{ce}}^{-\beta (I-100)}+b$$

Assuming the boundary conditions, when:

(7) $$I=100,\;\alpha =\alpha_{0}$$

When:

(8) $$I=800,\;\alpha =\alpha_{s}$$

Equation (6) can be rewritten as:

(9) $$\alpha =\alpha_{s}+(\alpha_{0}-\alpha_{s})e^{-\beta (I-100)},\;\left(100\leq I\leq 800\right)$$

**Figure 22:**
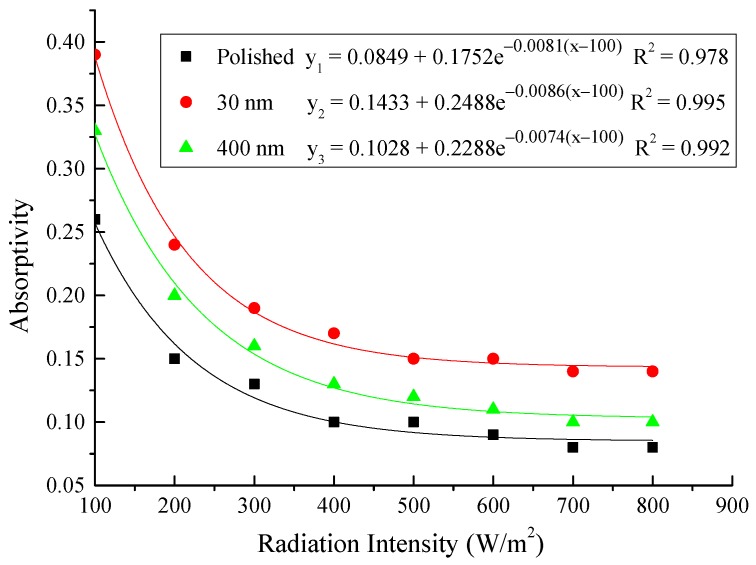
Correlation of the absorptivity with radiation intensity for the three testing rigs.

Then, the calculation results of the absorptivity of the three types of aluminum sheets were shown in Table 3, and the correlation of the absorptivity with the radiation intensity was presented in Figure 22. The exponential relation between the absorptivity and the radiation intensity was found for the three testing rigs that the increase of the radiation intensity had lead to the decrease of the absorptivity. The comparison between the testing results and the calculation results indicated that the exponential function could predict the absorptivity of the aluminum sheet under different irradiation within the acceptable error range.

### 4.3. Error Propagation Analysis

Affected by the accuracy class of the testing devices, the uncertainty range of the major measure quantities containing temperature and solar radiation would be considered. From Table 1, the uncertainty range of the temperature and solar radiation could be calculated [28].

(10) δT=0.2%×(200+50)=0.5(°C)

(11) $$\delta I=0.5\%\times (2000+0)=10({W/m}^{2})$$

Taking the polished aluminum sheet at the radiation intensity was 100 W/m^2^ for example (due that the maximum error would appear at this condition), the relative error of the heat absorbed and absorptivity could be calculated [29].

The relative error of all the testing condition about heat absorbed would be less than 10.6%.

The relative error of all the testing condition about absorptivity would be less than 14.6%.

**Figure 23:**
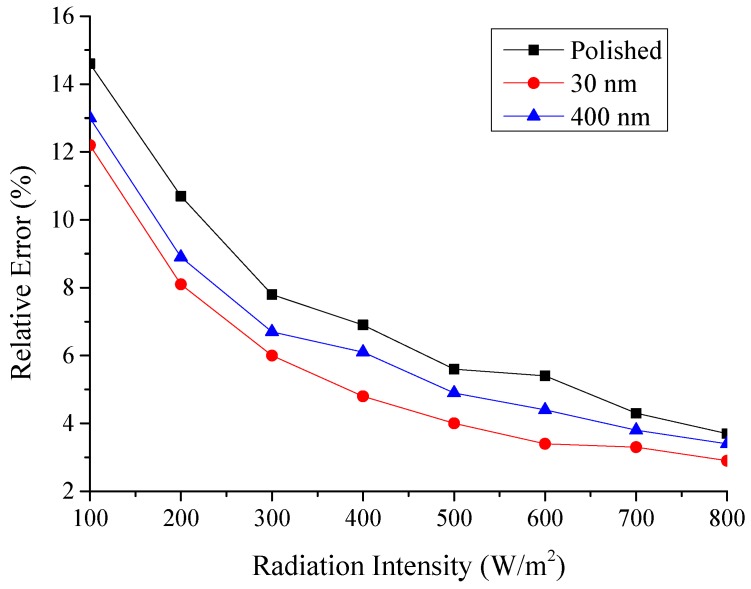
Relative error of the testing absorptivity with radiation intensity for the three testing rigs.

The relative error of the absorptivity for the other two sheets and at the other testing radiation intensity could be calculated by using the same method, the results were show in Figure 23.

Therefore, the error with this experimental could be acceptable from Figure 23.

## 5. Conclusions

In this paper, the nanoporous alumina sheets with the pore diameters of 30 nm and 400 nm were prepared by anodic oxidation method, and the SEM indicated that the nanopores evenly distributed on the surface of the aluminum sheets, which will be helpful in enhancing the stability of the aluminum surface and improving the solar absorption capability. In order to investigate the property of the nanoporous alumina sheet, the prepared aluminum sheets were compared with the conventional polished aluminum sheet and analyzed in the aspects of the instantaneous absorptivity, average absorbed heat and average absorptivity. The exponential correlation between the average absorptivity and the radiation intensity for the three testing rigs was also established. 

The results showed that the maximum instantaneous absorption efficiency of the nanoporous alumina sheet with 30 nm pore diameter was found at 0.92 when the radiation intensity was at 100 W/m^2^. The absorbed heat by the testing rigs increased with the increase of the radiation, while the average absorptivity decreased with the increase of the radiation. When the radiation intensity was 100 W/m^2^, the nanoporous alumina sheet with the 30 nm pore diameter had the highest average solar absorptivity of 0.39, which was 18% higher than that of the nanoporous alumina sheet with 400 nm pore diameter, and 50% higher than that of the polished aluminum sheet. The results indicated that the nanoporous alumina sheet performed better than the conventional polished aluminum sheet in the solar absorption property, and the pore size significantly influenced the solar absorptivity of the aluminum sheet. By error propagation analysis, the relative error of the average amount of heat absorption and the average absorptivity were acceptable.

## Figures and Tables

**Table 1:** Performance parameters of the testing devices.

Testing Device	Type	Performance Parameters
Solar radiation simulator	AHD2000	Wavelength range: 0.2–2 μm; power: 2000 W; luminous flux: 32,000 L m; light centre height: 123 mm; color temperature: 5700 K
Multichannel temperature monitor	AT4340	Sensor: K type thermocouple; measuring range: −50–200 °C; accuracy class: 0.2; power supply: 220 V ± 10%, 50 Hz ± 2%; environmental data range: temperature −20–70 °C, humidity 20%–90%
Solar radiation observation station	JTTF	Sensitivity: 7–14 mV/(kW·m^2^); spectral range: 0.3–3 μm; response time: ≤30 s; measuring range: 0–2000 W/m^2^; accuracy class: 0.5
Multichannel temperature and humidity detection system	PC-2WS	Temperature measurement range: −50–120 °C; temperature accuracy: ±0.2 °C; relative humidity measurement range: 0%–100%; relative humidity accuracy: ±2%

**Table 2:** Average instantaneous absorptivity for the testing rigs operated under different radiations.

**Radiation Intensity (W/m^2^)**		100	200	300	400	500	600	700	800
**Average Instantaneous Absorptivity**	Polished aluminum sheet	0.26	0.15	0.13	0.10	0.10	0.09	0.08	0.08
	Nanoporous alumina sheet with 30 nm pore diameter	0.39	0.21	0.18	0.17	0.15	0.15	0.14	0.14
	Nanoporous alumina sheet with 400 nm pore diameter	0.33	0.20	0.14	0.13	0.12	0.11	0.11	0.10

**Table 3:** Relative error of the calculated and testing absorptivity for the testing rigs operated under different radiation intensity.

Radiation Intensity (W/m^2^)	Polished Aluminum Sheet			Nanoporous Alumina Sheet with 30 nm Pore Diameter			Nanoporous Alumina Sheet with 400 nm Pore Diameter		
	Calculate Values	Testing Results	Relative Error	Calculate Values	Testing Results	Relative Error	Calculate Values	Testing Results	Relative Error
100	0.26	0.26	0	0.39	0.39	0	0.33	0.33	0
200	0.16	0.15	6%	0.25	0.21	16%	0.21	0.20	5%
300	0.12	0.13	−8%	0.19	0.18	5%	0.15	0.14	7%
400	0.10	0.10	0	0.16	0.17	−6%	0.13	0.13	0
500	0.09	0.10	−11%	0.15	0.15	0	0.11	0.12	−9%
600	0.09	0.09	0	0.15	0.15	0	0.11	0.11	0
700	0.09	0.08	11%	0.14	0.14	0	0.11	0.11	0
800	0.09	0.08	11%	0.14	0.14	0	0.10	0.10	0

## Nomenclature

*A*	area, m^2^
c_p_	specific heat capacity, J/(kg·°C)
*I*	solar radiation, W/m^2^
m	mass, kg
*Q*	amount of heat, J
*Q′*	instantaneous amount of heat, J
Δ*t*	average time difference, min
Δ*t′*	instantaneous time difference, min
*T*	temperature, °C
*α*	absorptivity
*α′*	instantaneous absorptivity
b, c, β, h	constant

## Subscripts

al	aluminum sheet
w	water
s	steady state
0	initial
t	total
δ	uncertainty range
